# A zero-gap silicon membrane with defined pore size and porosity for alkaline electrolysis†

**DOI:** 10.1039/d4se00515e

**Published:** 2024-06-09

**Authors:** Akash Raman, Sjoerd van der Werf, Cavit Eyövge, Miguel Angel Rodriguez Olguin, Stefan Schlautmann, David Fernández Rivas, Bastian Mei, Han Gardeniers, Arturo Susarrey-Arce

**Affiliations:** a Mesoscale Chemical Systems Group, MESA+ Institute for Nanotechnology, Faculty of Science and Technology, University of Twente P. O. Box 217 7500 AE Enschede The Netherlands a.raman@utwente.nl a.susarreyarce@utwente.nl; b Photocatalytic Synthesis Group, MESA+ Institute for Nanotechnology, Faculty of Science and Technology, University of Twente P. O. Box 217 7500 AE Enschede The Netherlands; c Technische Chemie, Ruhr-Universität Bochum Universitätsstr. 150 44801 Bochum Germany

## Abstract

Porous separators are a key component in alkaline water electrolyzers and are significant sources of overpotential. In this paper, porous silicon separators were fabricated by etching precise arrays of cylindrical pores into silicon substrates through lithography. Chemical stability of the silicon-based separators is ensured through the deposition of a silicon nitride layer. Platinum or nickel were vapor-deposited directly on the faces of the separator to complete a zero-gap configuration. Separator porosity (*ε*) was varied by changing the pore diameter and the pore spacing. These well-controlled porous silicon zero gap electrodes (PSi-ZGEs) were used to study the trade-off between separator resistance and gas-crossover at different porosities. It was found that separator resistances comparable to commercially used Zirfon UTP 500 were achieved at much lower *ε*. Gas crossover remained within the explosive limits for *ε* ≤ 0.15%. The PSi-ZGEs achieved stable performance at 100 mA cm^−2^ for 24 hours without significant surface damage in the alkaline electrolyte. In the broad perspective, the current work can pave the path for the development of ionomer-free separators for alkaline water electrolysis which rely on the separator geometry to limit gas-crossover.

## Abstract 2

Stable porous silicon based separators for alkaline water electrolysis with micron-sized cylindrical pores and low area resistance.

## Graphical abstract

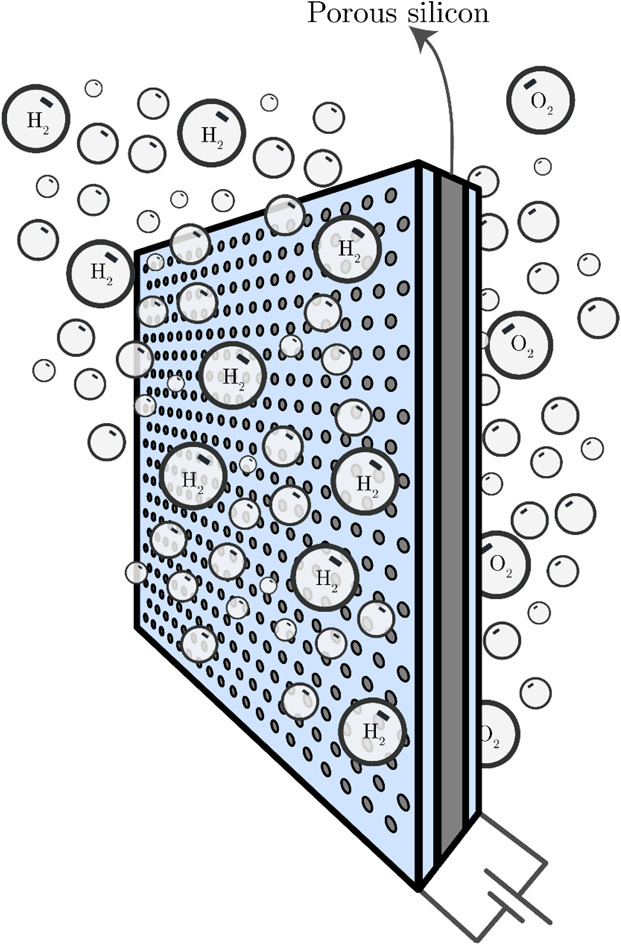

## 1 Introduction

Alkaline water electrolysis and proton exchange membrane electrolysis are the two most commercially deployed electrolysis technologies.^1^ Proton exchange membrane electrolyzers (PEMEs) typically offer higher efficiencies and operate at a higher current density than their alkaline counterparts. However, the efficiency of PEMEs degrades twice as fast as alkaline water electrolyzers (AWEs) – nullifying the advantage of their higher beginning-of-life efficiency.^2,3^ Furthermore, PEMEs require the use of expensive titanium-based components, iridium-based anodes, and platinum-based cathodes.^4,5^ AWEs on the other hand utilize cheaper non-platinum group metal (non-PGM) electrodes such as iron, cobalt, and nickel. In addition, PEMEs have more demanding water quality specifications. As a result, AWEs are typically cheaper to set up and operate.^6,7^ This makes AWEs more suitable for the large-scale water electrolysis plants needed to achieve gigawatt-scale production where a minimum load is generally assured.^8^ Improvements in AWE efficiency are crucial to reducing stack dimensions and, consequently, material costs.^9^

A key development in AWEs is the adoption of a zero-gap electrode architecture aimed at lowering the ohmic overpotential by reducing the inter-electrode distance and allowing the electrolyzer to operate at a higher current density. Zero-gap electrodes (ZGEs) are typically made by compressing two electrodes on either side of a porous separator. The inter-electrode distance in a ZGE configuration is equal to the thickness of the membrane or the separator. The ohmic resistance of the cell should therefore be approximately equal to the ohmic resistance of the separator alone.^10^ However, this is not the case for AWEs separators, such as Zirfon,^10–14^ where the ohmic resistance of the cell has been found to be almost twice the resistance of the separator alone.^15,16^ Contrary to the expectation that the adoption of a zero-gap configuration would reduce losses due to bubbles, it has been shown that the additional ohmic penalty in ZGEs arises from the formation of gas bubbles in the gap between the electrodes and the membrane – indicating the scope for further optimization of the electrode geometry and configuration.^17,18^ It has been proposed that a 0.2 mm gap is optimal in removing a large part of the bubble-induced overpotential in a ZGE configuration.^17^ The need to further reduce the resistance of porous separators has been identified as a key step in making AWEs competitive with PEMs in terms of efficiency and operating current density.^15^

For the same separator thickness, the migration path length of ions in the electrolyte decreases with increasing porosity and this leads to a lower ohmic overpotential. However, this comes at the cost of increased gas crossover another critical factor which limits the maximum current density of electrolyzers for safe operation.^19^ Gas crossover from one half-cell to the other can occur in two ways – due to the transfer of gas bubbles or due to the diffusion of dissolved gases. Resistance to bubble crossover strongly depends on the (average) pore size, with smaller pores being better at excluding gas bubbles.^8^ However, gas crossover due to the transport of dissolved gases is more strongly influenced by the (mean) diffusion path length between the two half-cells.

Both ions in the electrolyte, and dissolved gas molecules are orders of magnitude smaller than gas bubbles. Therefore, their transport across the separator is determined by the migration and diffusion path lengths respectively. As a result, the separator resistance, and the gas crossover due to the diffusion of dissolve gases is less sensitive to pore size. Other separator properties such as tortuosity play a key role in determining migration and diffusion across the separator. High tortuosity can increase the path length for ionic migration within the separator pores, increasing ohmic resistance but tortuous pores might be beneficial for gas crossover inhibition.

A better understanding of the effect of separator properties such as pore size and porosity on the separator resistance in ZGEs would be a step towards the development of better separators. This can be achieved by recreating a zero-gap membrane with well-defined and regular pores to eliminate the effects of tortuosity, and variations in porosity. Further, by depositing the electrode materials directly atop the separators, the effect of porosity and pore size can be studied in the absence of the previously observed^17^ losses associated with bubble nucleation between the electrode and the separator. In such a system, the resistivity of separators can be minimized by increasing the separator porosity, which essentially represents the percentage of the membrane volume filled with electrolyte.^20–22^

The combination of standard photolithography with techniques such as reactive ion etching and anodization can facilitate the micromachining of well-defined pores in silicon. These silicon-based micromachining approaches have previously been studied as alternatives to ionomeric membranes such as Nafion in microfluidic fuel cells,^19,23–30^ as separators in redox flow batteries,^31^ and for use in electrolyzers.^21^ However, the demonstration of such separators for use in alkaline water electrolysis with a pore diameter in the order of a few micrometers and a separator thickness in the tens of micrometers has not been achieved due to challenges associated with standard photolithographic techniques. Bosserez *et al.* produced a separator without ionomers with the necessary functional elements assembled in a photoelectrochemical cell (PEC) configuration.^20^ The etching of PEC elements like silicon in alkaline conditions did not allow long-term experiments in the aforementioned studies. In addition to that, the ohmic resistance of the PEC separator remained 1-fold higher than Zirfon – the separator of choice for commercial alkaline water electrolysis.^10–14^ Importantly, the H_2_ concentration at the anode of these devices was reported to be as high as ∼20% – far higher than the safety limits dictated by the explosion limits.

One way to minimize gas crossover is the deposition of ionomers inside the pores. A key drawback of ionomers is that they are typically incompatible with silicon-based monolithic microelectromechanical systems (MEMS) because they introduce additional stresses due to their volumetric expansion in electrolytes.^20,32,33^ Efforts, therefore, are needed to leverage photolithography to exploit the synergy between silicon-based separators and compact micromechanical systems. Unlike ionomers, porous silicon (PSi) has no charged surface moieties which enable conduction, *i.e.*, ionic conductivity is solely provided by the electrolyte filling the pores. Thus, unmodified PSi acts as a passive diaphragm like Zirfon. Several strategies to impart ion conductivity to porous silicon separators have also been considered. These include silanization to introduce charged surface groups on the pore walls^24,25^ and filling the pores with concentrated liquid electrolytes.^28,29^ Despite the inherent advantage of lower ohmic losses in silicon separators, which can be significantly thinner than commercial membranes, PSi-based devices exhibit unsafe gas crossover levels, severely limiting their applicability.

This paper reports the fabrication and analysis of stable, porous separators with micron-sized pores (2.4 μm, 4.1 μm and 7.9 μm), low ionic resistance, and low gas crossover. PSi separators with ordered and well-defined straight pores are fabricated using silicon photolithography. The deposition of a silicon nitride layer on the porous separator imparted chemical resistance, contributing to the stability of the separator in alkaline conditions. As a proof of concept, a zero-gap configuration has been obtained by depositing the electrocatalyst – platinum or nickel – directly on either face of the separator. It was found that the porosity, which is related to the pore size and spacing between the pores, has a significant effect on the ionic resistance of the membrane and the purity of the gas produced. The highest purity of the produced gases was obtained at the lowest porosity, while the lowest ionic resistance has been found at the highest porosity. From a broad perspective, the current work can pave the path for developing multi-stack ionomer-free systems that rely purely on the separator geometry applied for alkaline water electrolysis.

## 2 Materials and methods

### 2.1 Fabrication of the zero-gap electrodes

**Fig. 1:**
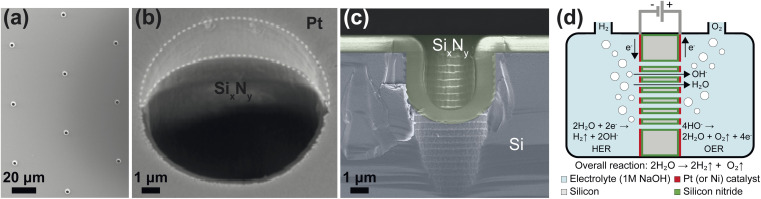
(a) A representative scanning electron microscopy (SEM) image showing pores of diameter *d*_p_ = 2.4 μm arranged in a triangular pitch configuration with a pore-to-pore spacing *s*_p_ = 75 μm. (b) A representative SEM image shows the extent of Pt coverage inside the pore is limited and the insulating Si_*x*_N_*y*_ keeps the two electrodes isolated from one another. (c) SEM close-up of the cross-section of an (intentionally) fractured pore. The silicon is shaded purple, and the Si_*x*_N_*y*_ regions are shaded green. (d) An illustration of the electrochemical cell with the perforated zero-gap membrane integrated. The silicon, platinum, and Si_*x*_N_*y*_ regions of the membrane are marked in gray, red, and green, respectively. The Si_*x*_N_*y*_ layer insulates the porous silicon and stabilizes the separator in the alkaline medium. The directions of the flow of electrons and ionic species are indicated with arrows.

The zero-gap electrodes with a porous silicon separator were fabricated from a silicon-on-insulator (SOI) wafer. First, a 40 μm thick, porous membrane was etched using reactive ion etching, which resulted in well-defined, cylindrical pores in a triangular pitch pattern. The porosity of the separator was varied by changing the pore diameter, *d*_p_ and the pore spacing, *s*_p_. A silicon nitride (Si_*x*_N_*y*_) layer was deposited on all silicon surfaces to ensure electrical insulation as well as the chemical stability in alkaline conditions. An SEM image of pores in the silicon substrate arranged in a triangular pitch pattern is shown in Fig. 1(a) after non-stoichiometric silicon nitride (Si_*x*_N_*y*_) deposition.

The zero-gap configuration was completed by vapor-depositing metal layers (either platinum or nickel) on either side of the silicon separator. Platinum and nickel were used as a proof-of-concept catalysts which could be readily deposited on the PSi. In both cases, metal vapor deposition was performed at an angle to ensure the pore openings remained unobstructed. This can be seen in Fig. 1(b) where the intrusion of the platinum layer into the pore opening is limited. Fig. 1(c) shows the close-up of the cross section of a single pore where the conformally deposited Si_*x*_N_*y*_ layer is visible. Fig. 1(d) shows a schematic of the substrates used in the study. The fabrication steps are further detailed in ESI Section S2.† An SEM image showing a wide view of a PSi-ZGE is shown in ESI Fig. SI9(a).†

### 2.2 Electrochemical cell and gas chromatography measurements

The zero-gap electrodes were placed in a custom-built electrochemical cell comprising two identical half-cells with the electrode in between, as shown in Fig. 1(d). The design of the custom electrochemical cell is explained in detail in ESI Section S3.† In all experiments, 1 M NaOH was used as the electrolyte in both half-cells. The half-cells were equipped with gas outlets which were connected to the gas chromatograph (GC) (see Fig. SI4†) and ports for reference, and auxiliary electrodes. First, a GC calibration curve was generated by mixing hydrogen, oxygen and the carrier gas helium, in varying proportions. This was done in order to verify the linearity of the response (see ESI Section S4†). In order to measure the gas crossover and the faradaic efficiency, 10 mA cm^−2^ was applied to the cell and the system was allowed to reach a steady-state for 40 min. It was found that the oxygen gas concentrations were much lower than expected – likely due to high humidity in the gas stream. As a result, gas purity and the faradaic efficiency were calculated on the basis of a baseline measurement where 100% purity was assumed (see ESI Section S4†).

### 2.3 Ionic resistance measurement

Galvano-dynamic current sweeps were used to determine the ionic resistance attributable to the separator. A four-electrode setup as shown in Fig. SI5† was used for these experiments. The reference electrodes were positioned ≈ 2 mm away from the center of the separator to ensure that only the potential drop across the separator was measured. Baseline measurements were made without a separator but with a Teflon spacer of equivalent thickness in order to account for the solution resistance. The increase in resistance from this baseline was therefore entirely attributable to the separator.

The electrochemical cell was set up with a PSi separator clamped between the two cell halves (see ESI Fig. SI5†). The four-electrode setup was completed with a pair of platinum auxiliary electrodes with a length of 3 cm. These platinum electrodes were placed in the two end caps and made leak-proof with an o-rings. The electrolyte solution of 1 M NaOH was prepared and filled into the electrochemical cell.

The four electrodes were connected to the potentiostat (Biologic SP-150) and current was applied between the two auxiliary electrodes while the potential was measured between the two reference electrodes. The current was swept from 0 mA cm^−2^ to ±10 mA cm^−2^ at a scan rate of ±1 mA s^−1^ respectively. An open circuit rest period of 30 s was maintained between the anodic and cathodic sweeps. The resulting voltage drop over the PSi separator was measured and the separator area resistance (*R*_s_) was calculated using Ohm's law after accounting for the baseline measurement. The conductivity of each electrolyte batch was measured with a pH/conductivity meter (Mettler Toledo S47 SevenMulti™) and a corrections were applied to account for variations in electrolyte conductivity. Experiments were done in triplicates for each type of PSi produced (see ESI Table SI1†). The experiments were also repeated with separators without the Pt or Ni catalyst to confirm that the deposition of the metal layers did not affect the ionic resistance of the separators.

### 2.4 Scanning electron microscopy images

Scanning electron microscopy images of samples were taken using a Zeiss MERLIN SEM microscope operated at 2 kV coupled with High-Efficiency Secondary Electron Detector (HE-SE2). Silicon separators coated with catalyst (Pt or Ni) were analyzed on top of conductive carbon tape.

## 3 Results and discussions

**Fig. 2:**
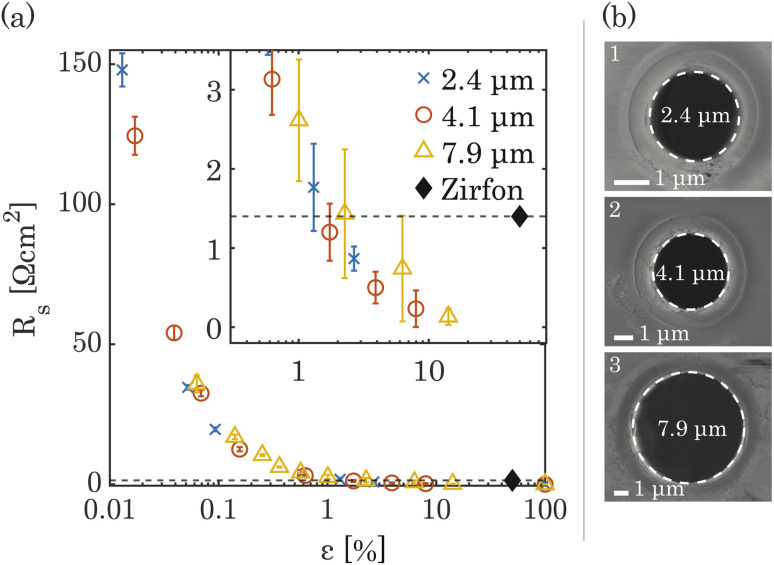
(a) The area resistances *R*_s_ of PSi-ZGE measured from galvano-dynamic current sweeps are shown on a logarithmic scale. The dashed horizontal line represents the separator resistance of Zirfon Perl UTP 500. The error bars represent one standard deviation about the sample mean. A magnified section of the plot is presented in the inset to provide a clearer view of the ionic resistances of the PSi-ZGE for *ε* > 1%. (b) SEM images of individual pores with *d*_p_ = 2.4 μm (1), *d*_p_ = 4.1 μm (2), and *d*_p_ = 7.9 μm (3).

The ionic resistance was calculated from the slope of potential and current obtained from the galvano-dynamic sweeps described in Section 2.3. The separator area resistance (*R*_s_) was calculated as the additional cell resistance introduced by the PSi-ZGE over the pure solution resistance measured without a separator between the two half-cells. Furthermore, the ionic resistance of bare PSi separators without deposited metal was found to be identical to that of a metal-coated PSi-ZGE confirming that the presence of the metal layers did not influence the ohmic drop across the separators. This observation is in agreement with the observations made from the SEM images (Fig. 1(b)), which show that the pores remain unobstructed by the deposited metal layers. Fig. 2 shows *R*_s_ as a function of the porosity of the separator (*ε*, in %). The data points of all pore diameters collapse onto a single curve indicating the fact that *ε* rather than *d*_p_ determines the ionic resistance of the separator. This can be explained by the fact that the charge-carrying ions are orders of magnitude smaller than the pore diameters considered in this study (ESI Table SI1†).

The ionic resistance of the Zirfon sample at room temperature was measured in the same holder using the aforementioned protocol and found to be 1.4 Ω cm^2^. The zoomed-in inset in Fig. 2(a) shows that PSi-ZGE with *ε* > 1% can achieve lower ionic resistances than Zirfon (*ε* ∼ 50%). One factor that contributes to the lower ionic resistance of PSi-ZGEs (thickness 40 μm) is that they are ∼12 times thinner than the Zirfon separator (thickness 500 μm). Furthermore, ion transport across separators is affected by the tortuosity of the separator^34^ and Zirfon has a porous network characterized by high tortuosity, while PSi-ZGEs have well-defined, cylindrical pores as seen in Fig. 2(b). The tortuosity of the PSi-ZGEs is 1 since the length of the pores is equivalent to the thickness of the separator. On the other hand the tortuosity of Zirfon has been reported to lie between 1.5 and 2.8.^35^ Therefore, the migration path-length for ions across the Zirfon separator is much longer on account of the greater thickness as well as the greater tortuosity. These two factors account for the lower ionic resistances of PSi-ZGE in comparison to the Zirfon separator.

Note that the vapor-deposited metal electrodes, *i.e.*, platinum or nickel, were not connected to the potentiostat during ionic resistance measurements in order to avoid bubble generation on the PSi-ZGE. The PSi-ZGEs were compared with uncoated Zirfon separators. This is because metal deposition on Zirfon would have changed the separator porosity.

**Fig. 3:**
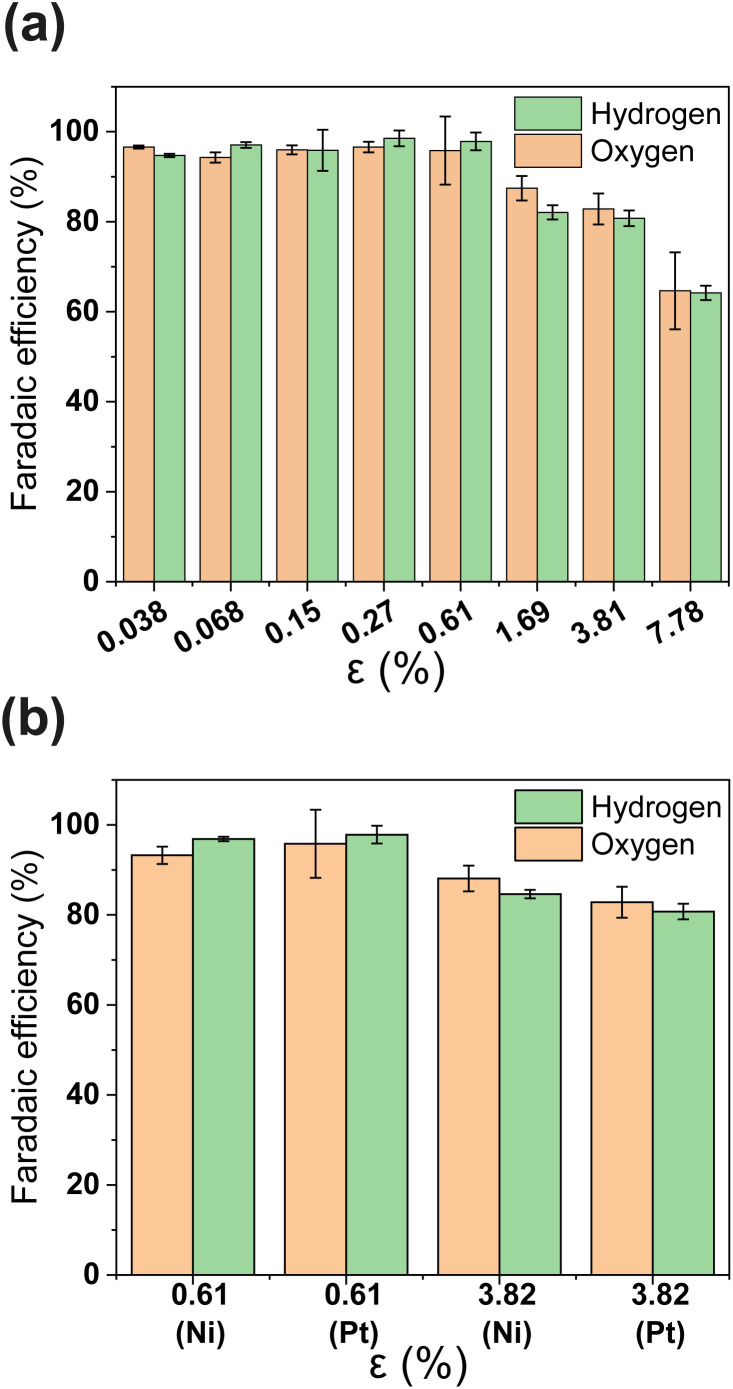
(a) The faradaic efficiency for H_2_ and O_2_ of PSi-ZGEs with a pore diameter of 4.1 μm at a constant current density of 10 mA cm^−2^ is shown for separators of different porosities. (b) The faradaic efficiency for H_2_ and O_2_ with Pt, and Ni electrocatalysts are compared at *ε* = 0.61% and *ε* = 3.82% porosity. All error bars indicate one standard deviation about the sample mean.

PSi-ZGEs with *d*_p_ = 4.1 μm were used for further analysis since they were expected to yield a trade-off between ionic resistance and gas-crossover. The faradaic efficiency (FE) of PSi-ZGE is shown in Fig. 3(a) and (b) as a function of porosity. The methodology used to estimate the H_2_ and O_2_ concentration and FE are described in Section 2.2 and ESI Section S4.† Experiments to calculate the FE were performed with either a 200 nm Pt or Ni electrode deposited on the surfaces of the separators (see Fig. 3(b)). Ni has the advantage of being a more stable electrocatalyst for OER than Pt and represents a low-cost catalyst ideal for larger-scale zero-gap membranes for alkaline electrolysis.^36^

It can be seen from Fig. 3(a) that the FE is ∼100% for *ε* ≥ 0.61 calculated on the basis of either hydrogen or oxygen. Increasing the porosity further leads to a decrease in FE. A FE of ∼60% was measured for the greatest porosity considered in the study – *ε* = 7.78%. The decrease in FE with increasing *ε* can be attributed to increased O_2_ crossover and subsequent oxygen reduction at the cathode. It is important to note that electrocatalyst composition does not have an apparent effect in Fig. 3(b)*i.e.*, the FE of PSi-ZGE with platinum and nickel are approximately equal when compared at the same *ε*.

**Fig. 4:**
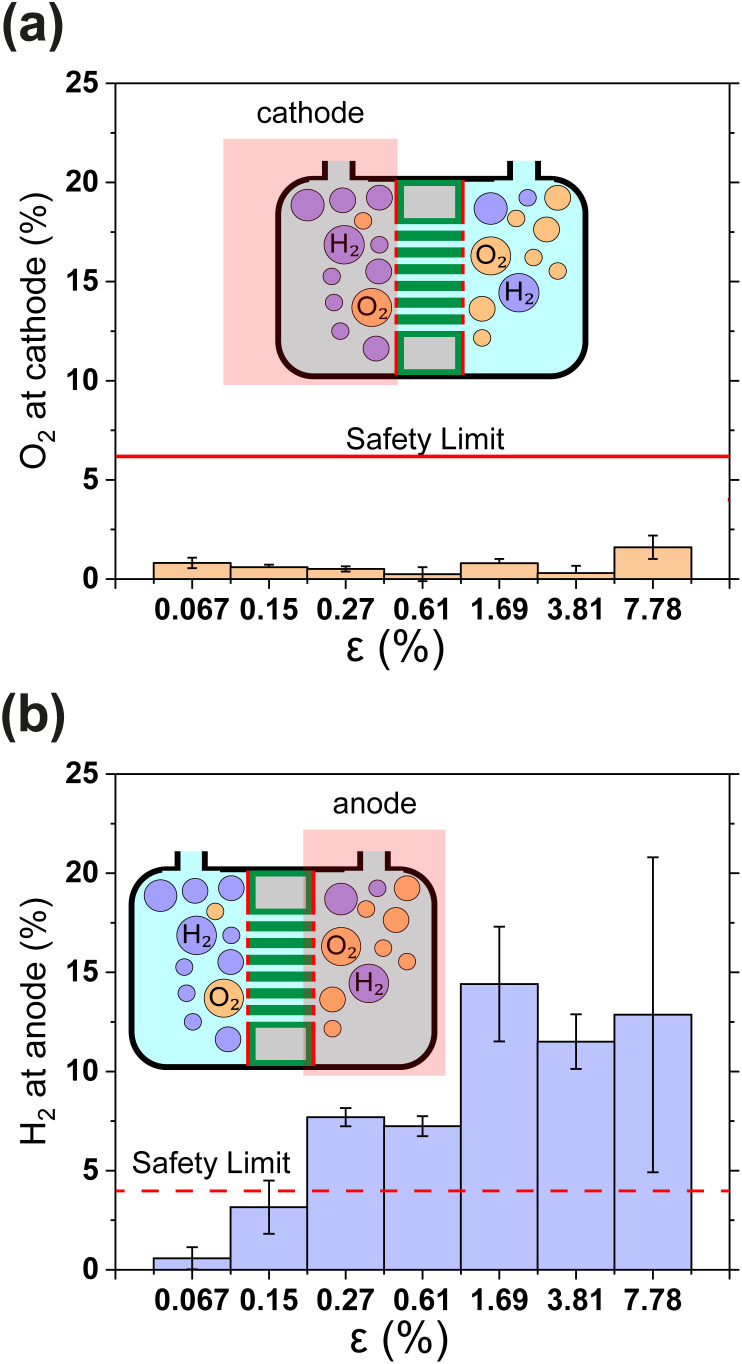
(a) and (b) Show the relative concentrations of O_2_ in the cathodic gas outlet and H_2_ in the anodic gas outlet streams. In (a) and (b) outlet pressure was 1.3 bar. The flammability limits of H_2_ and O_2_ mixtures are indicated as horizontal lines.^38^ In panels (a) and (b) gas concentrations are expressed as volume percentages. All error bars indicate one standard deviation about the sample mean.

Gas crossover is studied in Fig. 4(a) and (b) which show the oxygen and hydrogen crossovers as functions of *ε*. Fundamentally, the permeability of the gas separator is related to the bubble point pressure. In other words, it is the pressure required to blow air through a liquid-filled separator.^37^ For a high bubble point pressure, smaller pore sizes are beneficial to decrease permeability. Therefore, *d*_p_ and *ε* are decisive resistance parameters for gas-crossover. It can be seen that the O_2_ concentration at the cathode remains well below the explosion limit (<6%) for all porosities.^38^ This is in line with expectations since the oxygen reduction reaction is likely to occur easily at the metallic cathode. On the other hand, Fig. 4(b) shows that hydrogen crossover increases with increasing porosity and the hydrogen concentration in the anode gas stream rises beyond the lower explosive limit of 4% for *ε* > 0.15%. These measurements were intentionally performed in a batch electrolysis cell without electrolyte convection. Gas purity values obtained in this manner represent an extreme case such as the failure of circulation systems in an electrolyzer. The values reported in Fig. 4 therefore represent the upper bound. The introduction of convection is expected to dramatically improve the evacuation of gas from the electrode surface and lead to lower gas crossover.

The results in Fig. 4 show that H_2_ permeability to the anode is a latent challenge related to the porosity and pore dimensions. With an average pore diameter of 130 nm, thickness of 500 μm and porosity close to 50%, H_2_ permeability in Zirfon UTP 500 is relatively high.^39^ Thus, it is not surprising that for 40 μm thin PSi-ZGEs with *ε* > 0.15%, the hydrogen concentration at the anode exceeds the safety limit. Nonetheless, PSi-ZGEs with *ε* = 0.067% and *ε* = 0.15% show promise from a safety standpoint.

Silicon etches at a finite rate in strongly alkaline media which would lead to the failure of the PSi separator.^20,40^ However, our approach reduces the etching rate by incorporating a silicon nitride layer to the PSi followed by Pt. The stability of a platinum-coated PSi-ZGE (porosity 0.61%) is assessed at a constant nominal current density of 30 mA cm^−2^ for 14 h and 100 mA cm^−2^ for a continuous 24 hours to demonstrate our approach. Although the electrode layer in the platinum-coated PSi-ZGE is planar, it remains adhered to the PSi-ZGE after 24 h. SEM images in ESI Section S8† show the retention of the platinum layer but with an increase in roughness after 24 h, along with some openings in the Pt layer.

**Fig. 5:**
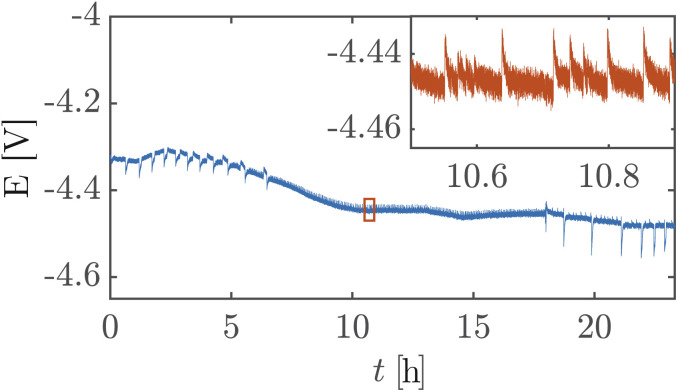
Variation of cell voltage with time recorded during a chronopotentiometry experiment performed with a platinum-coated PSi-ZGE with a pore size of 4.1 μm and porosity of 0.61% at a constant current of 100 mA cm^−2^ shows that PSi-ZGE does not materially degrade over the 24 hours. The fluctuations in potential due to bubble evolution are made clearer in the inset.

Fig. 5 shows a high cell potential of ∼4.5 V between the anode and the cathode for 100 mA cm^−2^. This is much larger than the values commonly reported in the literature for ZGEs^21^ even when accounting for the overpotentials arising from the use of Pt as the anode. Additionally, there is a noticeable increase in the cell potential in the first 10 hours of operation. This can be attributed to the build-up of a pH difference between the two half-cells^33^ and an increase in concentrations of the dissolved gases in the electrolyte. An explanation for such a large cell potential are ohmic losses associated with bubble generation and accumulation at the electrode.^41^ The periodic fluctuations seen in the inset in Fig. 5 can be attributed to periodic bubble evolution at the electrode surface.^42^ For a constant rate of gas generation at the electrode, *i.e.*, a constant current, it is possible to estimate the average departure size of bubbles from the electrode based on the frequency of fluctuations in the cell potential (see ESI Section S6†). Peak analysis of the 24 hour chronopotentiometry data yields a mean peak frequency of ∼0.7 Hz. For a constant current density of 100 mA cm^−2^, this frequency corresponds to a bubble diameter of ≈30 mm. While this figure is an overestimation because the algorithm used to detect peaks relies on finding local maxima in the *E vs. t* data, and only prominent peaks are detected by the algorithm. Nonetheless, this analysis confirms the buildup of large gas slugs inside the electrochemical cell which periodically bubble up towards the gas outlets.

**Fig. 6:**
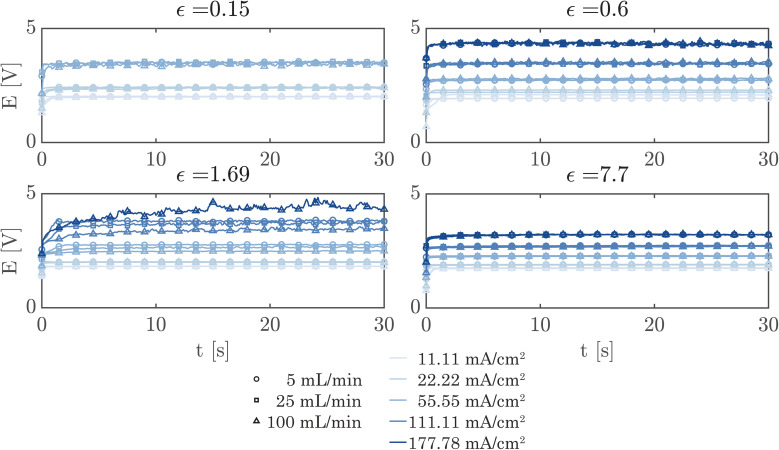
Potentiograms of PSi-ZGEs of different porosities at different applied currents and electrolyte flow rates. The curve colors represent the applied currents and the symbols represent the electrolyte flow rate (see common legend). Symbols are undersampled for better readability. Curves corresponding to the same current density but different flow rates lie close to one another. This indicates that the cell potential does not decrease with increasing flow rate unless a mass transfer limiting current density is approached.

A simple flow cell, as described in ESI Section S7,† was used to further demonstrate the performance of the PSi-ZGEs. Currents from 11.11 mA cm^−2^ to 177.78 mA cm^−2^ were applied and the potential was measured over 30 second periods at electrolyte flow rates of 5, 25 and 100 mL min^−1^. A syringe pump (Harvard Apparatus PHD 2000) was used for all flow experiments. Fig. 6 shows the potentiograms at different flowrates. Fig. 6 shows that while the introduction of electrolyte convection decreases the cell potential in comparison to Fig. 5, the flow rate does not significantly affect the cell potential for the values considered in the study.

**Fig. 7:**
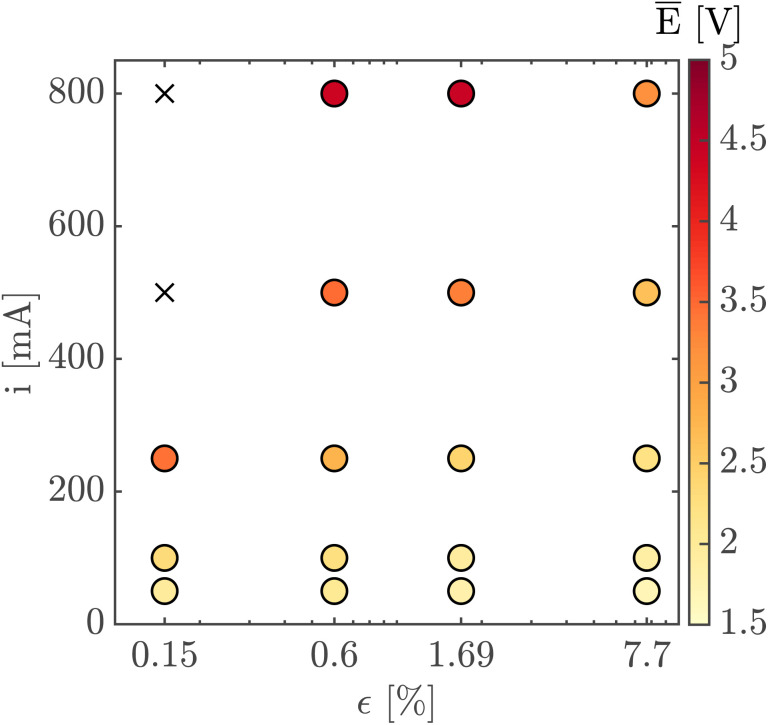
The average cell potential is shown for different currents and PSi-ZGE porosities at an electrolyte flow rate of 100 mL min^−1^. The color of the points indicates the magnitude of the average potential (see colorbar). The black crosses indicate combinations of current and porosity that were not possible to achieve because the cell potential exceeded the 5 V limit imposed on the potentiostat.

Finally, Fig. 7 shows the average cell potential at different applied currents and separator porosities at an electrolyte flowrate of 100 mL min^−1^. The data from Fig. 7 is tabulated in ESI Table SI2.† A potential limit of 5 V was applied to all experiments and experiments for separators with *ε* ≤ 0.15% was not possible for current densities above 55.55 mA cm^−2^. These data points are represented by crosses in Fig. 7. As expected, the cell potential increases with increasing current and decreases with increasing separator porosity. Due to the design of the flow cell, the exposed electrode area was 450 mm^2^. Therefore, the highest currents 500 mA and 800 mA correspond to current densities of 111.11 mA cm^−2^ and 177.77 mA cm^−2^ respectively. As expected, the introduction of forced convection alleviates the high potential seen in Fig. 5 in the absence of electrolyte convection.

The novel electrode architecture presented in this manuscript has several avenues for further optimization which were beyond the scope of this proof-of-concept study. The performance of PSi-ZGEs are expected to improve significantly with the use of better electrocatalysts.^43^ Furthermore, the variation in the surface properties, and stability of the electrocatalyst layer can be optimized by studying the effect of the parameters of the deposition step or by comparing other deposition methods. Finally, although the thickness of the Si was not studied in detail our experimental design and the structure of the PSi-ZGEs (cylindrical pores without interconnections) enables direct extrapolation to substrates of other thicknesses and an optimum between the mechanical properties of the separator and the ionic resistivity can be found.

## 4 Conclusion

The PSi-ZGE device presented in this work features two key improvements over previous monolithic porous silicon-based (photo)electrodes which were discussed in the introduction. Firstly, the conformal deposition of a silicon nitride layer imparts stability to these PSi-ZGEs under alkaline conditions. Secondly, the hydrogen crossover in the PSi-ZGEs in this work are shown to drop below the lower explosive limit for hydrogen for *ε* ≤ 0.15% without the use of ionomers. The absence of ionomeric fillings which undergo volume expansion, ensures the compatibility of these PSi-ZGEs with MEMS devices.

It was found that PSi-ZGEs have comparable ionic resistance to Zirfon UTP 500 at a much lower porosity. In fact, for *ε* > 1% PSi-ZGEs exhibit separator resistances lower than that of Zirfon UTP 500. Crossover of O_2_ into the cathodic chamber was observed to be well below the safety limit for all porosities considered in the study. On the other hand, the crossover of hydrogen remains within the lower explosive limit for *ε* ≤ 0.15%. The gas crossover was measured in the absence of forced convection and the gas purity values are meant to represent a worst-case scenario such as in the event of a circulation failure in an electrolyzer. In addition, the gas crossover measured in the absence of convection represents the separator's inherent ability to inhibit transport between the two half-cells.

Subsequently, the stability of the separators was tested by operating them at 100 mA cm^−2^ over a 24 hour period. SEM images showed a minor increase in the surface roughness of the Pt catalyst film. Finally, it is shown that current densities as high as 177 mA cm^−2^ can be reliably achieved in a flow configuration.

The PSi-ZGEs are versatile platforms that can be produced with a range of porosities, and pore sizes. Furthermore, the PSi-ZGE design used in this paper is electrocatalyst agnostic *i.e.*, a variety of electrode materials may be deposited on the surfaces of the separator. This is of particular interest in light of efforts to reduce the CRM-intensity of electrodes because non-PGM electrode materials can be adopted on this platform with relative ease. Such devices can be useful platforms for compact, small-scale alkaline water electrolyzers for miniaturized systems and for off-grid applications.

## Author contributions

A. R.: data curation, formal analysis, investigation, methodology, software, visualization, writing – original draft, writing – review & editing S. v. d. W.: data curation, formal analysis, investigation, methodology, software, visualization, writing – original draft, writing – review & editing. C. E.: investigation, methodology, visualization M. A. R. O.: investigation, methodology, visualization S. S.: methodology, writing – review & editing. D. F. R.: conceptualization, funding acquisition, methodology, supervision, writing – review & editing. B. M.: methodology, supervision, resources, writing – review & editing. H. G.: conceptualization, funding acquisition, methodology, supervision, writing – review & editing. A. S. A.: conceptualization, methodology, resources, supervision, writing – review & editing.

## Conflicts of interest

The authors declare that they have no known competing financial interests or personal relationships that could have appeared to influence the work reported in this paper.

## Supplementary Material

SE-008-D4SE00515E-s001

## References

[cit1] I. E. Agency , Global Hydrogen Review 2021, 2021

[cit2] David M., Ocampo-Martínez C., Sánchez-Peña R. (2019). J. Energy Storage.

[cit3] Felgenhauer M., Hamacher T. (2015). Int. J. Hydrogen Energy.

[cit4] Gago A. S., Lettenmeier P., Stiber S., Ansar A. S., Wang L., Friedrich K. A. (2018). ECS Trans..

[cit5] Marini S., Salvi P., Nelli P., Pesenti R., Villa M., Berrettoni M., Zangari G., Kiros Y. (2012). Electrochim. Acta.

[cit6] Brauns J., Turek T. (2020). Processes.

[cit7] Schmidt O., Gambhir A., Staffell I., Hawkes A., Nelson J., Few S. (2017). Int. J. Hydrogen Energy.

[cit8] Schalenbach M., Zeradjanin A. R., Kasian O., Cherevko S., Mayrhofer K. J. (2018). Int. J. Electrochem. Sci..

[cit9] Phillips R., Dunnill C. W. (2016). RSC Adv..

[cit10] de Groot M. T., Vreman A. W. (2021). Electrochim. Acta.

[cit11] Vermeiren P., Adriansens W., Moreels J. P., Leysen R. (1998). Int. J. Hydrogen Energy.

[cit12] Vermeiren P. , Adriansens W., Moreels J. P. and Leysen R., Hydrogen Power: Theoretical and Engineering Solutions, Dordrecht, 1998, p. 179–184

[cit13] Vermeiren P., Moreels J. P., Claes A., Beckers H. (2009). Int. J. Hydrogen Energy.

[cit14] Amores E., Sánchez-Molina M., Sánchez M. (2021). Results Eng..

[cit15] Schalenbach M., Tjarks G., Carmo M., Lueke W., Mueller M., Stolten D. (2016). J. Electrochem. Soc..

[cit16] Kraglund M. R. , PhD thesis, Department of Energy Conversion and Storage, Technical University of Denmark, 2017

[cit17] Haverkort J. W., Rajaei H. (2021). J. Power Sources.

[cit18] Nagai N. (2003). Int. J. Hydrogen Energy.

[cit19] Chu K.-L., Shannon M. A., Masel R. I. (2007). J. Micromech. Microeng..

[cit20] Bosserez T., Geerts L., Rongé J., Ceyssens F., Haussener S., Puers R., Martens J. A. (2016). J. Phys. Chem. C.

[cit21] Naito T., Shinagawa T., Nishimoto T., Takanabe K. (2022). ChemSusChem.

[cit22] Stojadinovic J., Wiedenmann D., Gorbar M., Mantia F. L., Suarez L., Zakaznova-Herzog V., Vogt U. F., Grobety B., Zuttel A. (2012). ECS Electrochem. Lett..

[cit23] Fang A. , PhD thesis, Cornell University, 2005

[cit24] Pichonat T., Gauthier-Manuel B. (2006). Fuel Cells.

[cit25] Pichonat T., Gauthier-Manuel B. (2005). J. Micromech. Microeng..

[cit26] Curlier P. , Bergamasco J.-L., Pichonat T., Marechal M., Gauthier-Manuel B. and Sanchez J.-Y., US Pat., US20040197613A1, 2004

[cit27] Yu Z., Zheng D., Zhang K., Yang T., Chen Y., Li X. (2017). Microsyst. Technol..

[cit28] Gold S., Chu K.-L., Lu C., Shannon M. A., Masel R. I. (2004). J. Power Sources.

[cit29] Chu K.-L., Gold S., Subramanian V., Lu C., Shannon M., Masel R. (2006). J. Microelectromech. Syst..

[cit30] Chu K.-L., Shannon M. A., Masel R. I. (2006). J. Electrochem. Soc..

[cit31] Carothers D. , Michaelson J. D., Redford R. G. and Roveda J. M., AU Pat., AU2020272134A1, 2021

[cit32] Trompoukis C., Abass A., Schüttauf J. W., Bosserez T., Rongé J., Lauwaert J., Martens J. A., Baets R. (2018). Sol. Energy Mater. Sol. Cells.

[cit33] Vijselaar W. J. C., Perez-Rodriguez P., Westerik P. J., Tiggelaar R. M., Smets A. H. M., Gardeniers H., Huskens J. (2019). Adv. Energy Mater..

[cit34] Tjaden B., Cooper S. J., Brett D. J., Kramer D., Shearing P. R. (2016). Curr. Opin. Chem. Eng.c.

[cit35] Rodríguez J., Palmas S., Sánchez-Molina M., Amores E., Mais L., Campana R. (2019). Membranes.

[cit36] Colli A. N., Girault H. H., Battistel A. (2019). Materials.

[cit37] Vermeiren P., Leysen R., Beckers H., Moreels J. P., Claes A. (2008). J. Porous Mater..

[cit38] Safety Standard for Hydrogen and Hydrogen Systems: Guidelines for Hydrogen System Design, Materials Selection, Operations, Storage and Transportation, NASA Technical Report NASA-TM-112540, 1997

[cit39] In Lee H., Dung D. T., Kim J., Pak J. H., Kim S. k., Cho H. S., Cho W. C., Kim C. H. (2020). Int. J. Energy Res..

[cit40] Shah I. A., van Enckevort W. J. P., Vlieg E. (2010). Sens. Actuators, A.

[cit41] Angulo A., van der Linde P., Gardeniers H., Modestino M., Fernández Rivas D. (2020). Joule.

[cit42] Raman A., Peñas P., van der Meer D., Lohse D., Gardeniers H., Fernández Rivas D. (2022). Electrochim. Acta.

[cit43] Anwar S., Khan F., Zhang Y., Djire A. (2021). Int. J. Hydrogen Energy.

