# Supplementary material for: A zero-gap silicon membrane with defined pore size and porosity for alkaline electrolysis
Source: Sustain Energy Fuels. 2024 Jun 9;8(15):3296–303. doi: 10.1039/d4se00515e (PMC11265265; doi:10.1039/d4se00515e)
Supplement: SE-008-D4SE00515E-s001 [file SE-008-D4SE00515E-s001.pdf]

Electronic Supplementary Material (ESI) for Sustainable Energy & Fuels.
This journal is © The Royal Society of Chemistry 2024

Supplementary Information to "A zero-gap silicon membrane with defined pore size and porosity for alkaline electrolysis"

Akash Raman[a,*,1], Sjoerd van der Werf[a,1], Cavit Eyövge[a], Miguel Angel Rodriguez Olguin[a], Stefan Schlautmann[a], David Fernández Rivas[a], Bastian Mei[b,c], Han Gardeniers[a], Arturo Susarrey-Arce[a,*]

[a] *Mesoscale Chemical Systems Group, MESA+ Institute for Nanotechnology, Faculty of Science and Technology, University of Twente, P.O. Box 217, 7500 AE Enschede, The Netherlands.*

[b] *Photocatalytic Synthesis Group, MESA+ Institute for Nanotechnology, Faculty of Science and Technology, University of Twente, P.O. Box 217, 7500 AE Enschede, The Netherlands.*

[c] *Technische Chemie, Ruhr-Universität Bochum, Universitätsstr. 150, 44801 Bochum.*

[*] *Corresponding author*

Email addresses: a.raman@utwente.nl (Akash Raman), a.susarreyarce@utwente.nl (Arturo Susarrey-Arce)

[1] *These authors contributed equally to this work.*

S1 Porosity calculation

Porosity is the fraction of the total volume of the separator that is accessible to the electrolyte. Since the pores in our separators are cylindrical and arranged in a regular triangular grid, the porosity can be calculated as the percentage of the surface area occupied by pores. This reduces the ratio of the cross-sectional area of one pore and the unit hexagon (see Fig. SI 1) whose inradius is equal to the pore spacing. Therefore, porosity ϵ can be expressed as,

$$\epsilon = \frac{\text{Pore cross-sectional area}}{\text{Area of unit hexagon}} = \frac{\pi\left(\frac{d_p}{2}\right)^2}{\sin\left(\frac{\pi}{3}\right)s_p^2} \times 100\% \tag{1}$$

where d_p is the pore diameter, and s_p is the pore spacing.

s_p (µm) → d_p (µm) ↓	14	20	30	50	75	100	125	150	200	300
2.4	2.66	1.3	0.58	0.2	0.09	0.05	-	0.02	0.01	0.006
4.1	7.77	3.81	1.69	0.6	0.27	0.15	-	0.06	0.03	0.01
7.9	-	14.15	6.28	2.26	1	0.56	0.36	0.25	0.14	0.06

Table SI 1: Porosity calculated as the ratio of the cross-sectional area of a pore and the area of a unit hexagon for combinations of pore spacings s_p and pore diameters d_p considered in this study. Note that the d_p and s_p were measured from SEM images taken after Si_xN_y and electrocatalyst (Pt or Ni) deposition.

S2 Silicon separator fabrication

The porous silicon membranes were fabricated from silicon-on-insulator (SOI) wafer from Si-Mat of 100 mm in diameter. The SOI wafer consists of a 500 μm thick Si handle layer, a 1 μm insulating buried oxide (BOX) layer, and a 40 μm thick Si device layer. Nine porous silicon (PSi) chips per wafer, each 24×24 mm^2 were fabricated. The resulting pore spacing and pore diameters are listed in Table SI 1.

The fabrication commences over the device layer. The device layer is spin-coated with photoresist (Olin OiR 907-17, 4000 rpm, 30s) and baked at 120 °C for 60 s. The photoresist is patterned with a mask and developed in OPD 4262 for 45 s. In this case, three separate mask designs with different pore diameters (4 μm, 6 μm, and 10 μm) and pore spacing were used in the study. After development, the pores are etched in a plasma etcher (5 μm/min, Plasma Pro 100 Estrelas), resulting in pores of depths similar to the device layer (i.e., 40 μm). Later the handle layer is etched at 20 μm/min in the silicon plasma etcher (Plasma Pro 100 Estrelas). The BOX layer is used as a protective layer during the etching steps of the device layer and handle layer. After the BOX layer is removed with HF both sides of the silicon membrane are open and ready for subsequent processing.

A Si_xN_y layer was deposited using LPCVD over the perforated silicon spacer to protect the silicon separator from the alkaline electrolyte. The electrocatalyst (Pt or Ni) was evaporated at an angle of 10deg with a Balzers BAK 600 with a crucible E Gun. The angled deposition ensured that the metal was not deposited inside the pores and prevented short-circuiting between the two electrodes. A Tantalum adhesion layer of 30 nm (0.15 nm/s, 220 mA) was deposited and followed by a 200 nm Pt (0.4 nm/s, 250 mA) or Ni (0.3 nm/s,

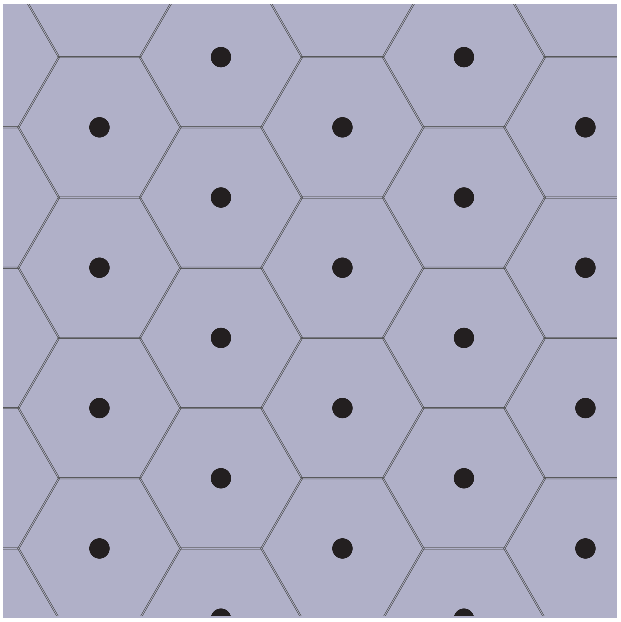

Figure SI 1: A representation of the hexagon associated with each pore. The pores on the separator are arranged in a triangular grid, with each pore occupying the center of a unit hexagonal cell.

120 mA). The base pressure was in the range of 5×10^{-7} mbar and the process pressure at 2×10^{-6} mbar. The diameter of the pores decreases from 4 µm, 6 µm, and 10 µminitially to 2.4 µm, 4.1 µm, and 7.9 µm. The final, active zero-gap electrode is a circular region of 2 cm diameter at the center of each chip resulting in an effective surface area of 3.14 cm^2 per substrate (see Fig. SI 2).

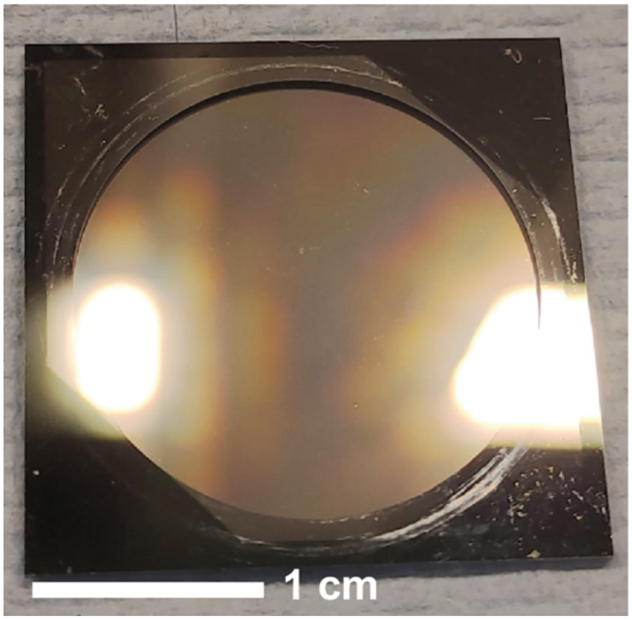

Figure SI 2: A camera image of a porous silicon separator sample with platinum. The platinum covered region appears lighter in the image.

S3 Quiescent studies cell design

The electrochemical cell comprises two cylindrical halves machined out of PEEK Fig. S3. The ZGE was placed between the two cells and is made leak-proof by O-rings on either side. The electrochemical cell was assembled by threading three long bolts through one compartment, placing the PSi electrode, placing the right half compartment, and fastening the bolts with wingnuts. The reference electrodes were inserted into the cell with PTFE (polytetrafluoroethylene) tape wound around them to create a water-tight seal.

The electrolyte (1M NaOH) was prepared, cooled to room temperature, and injected into the cell with the syringe. Next, the bubblers were attached. The fluidic connections were made using PTFE tubing and 1/4-28 standard port flat-bottom connectors. The bubblers were connected to the carrier gas inlet, where a mass flow controller (MFC) was used to regulate the inlet flow rate. Then the gas dryers were filled with fresh silica gel and connected to the electrochemical cell output (see Fig. SI 4)). Finally, the gas dryers are connected to the gas chromatograph (GC) with a limiting MFC.

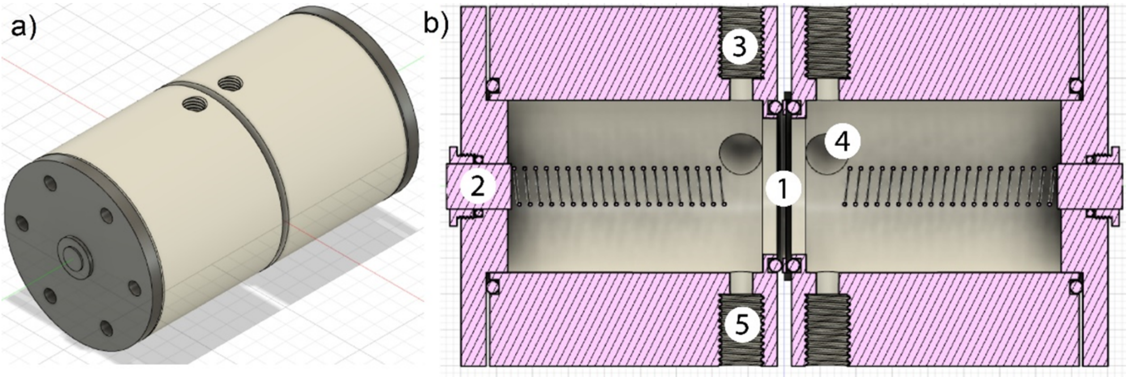

Figure SI 3: (a) A three-dimensional rendering of the custom-built electrochemical cell (b) A cross-sectional view of the electrochemical cell. The porous silicon zero-gap electrode (1) is placed between the two half-cells and held between two Viton o-rings. Platinum coil electrodes (2) were inserted into holes in both half-cells and were used to measure the ionic resistance of the separators. These holes were plugged during gas-evolution studies. Standard calomel reference electrodes were inserted in either cell compartment through dedicated ports (4) completing a 4-electrode cell. During gas evolution studies, the gas produced in the cell is collected from outlets (3) at the top of the cell, dried in silica gel-filled dryers and passed to the gas chromatograph for crossover measurements. The electrolyte is filled into the cell compartments from an additional port (5) which is closed during experiments.

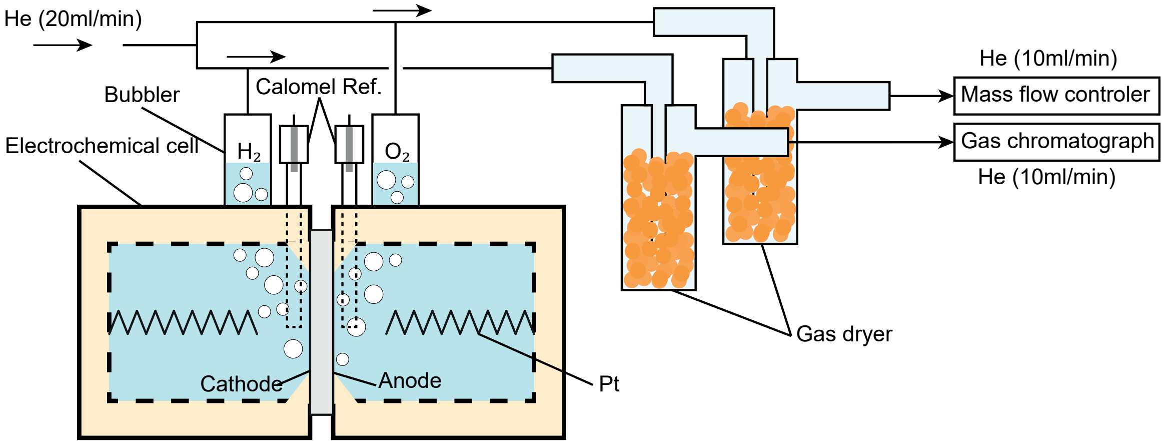

Figure SI 4: A schematic representation of the experimental set-up used to measure the gas-crossover and Faradaic efficiency of PSi-ZGEs.

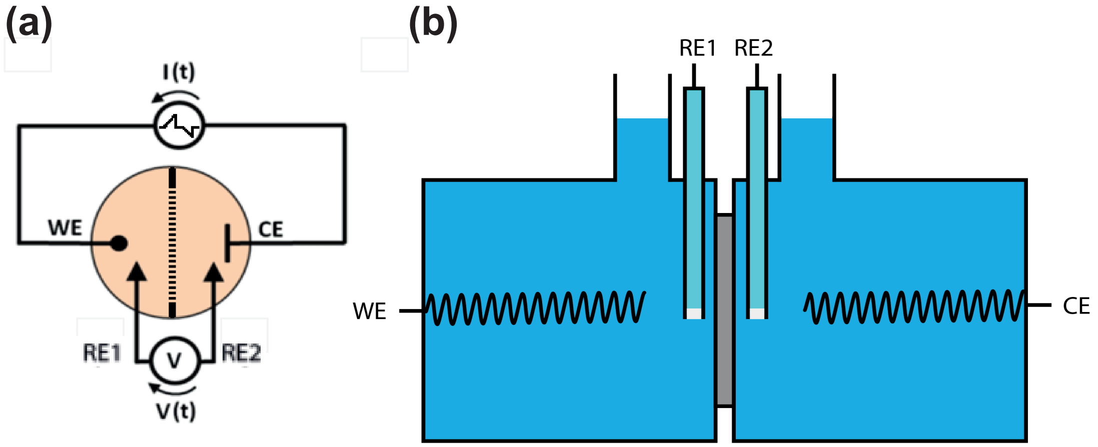

Figure SI 5: Illustration of the four electrode set-up used for ionic resistance measurements.

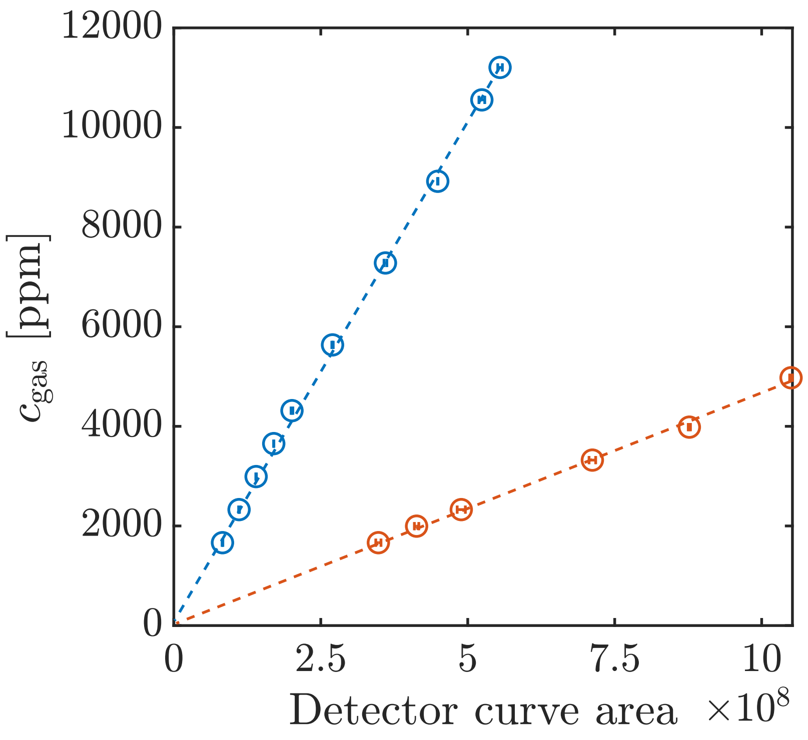

Figure SI 6: The calibration curves measured with the gas chromatograph for hydrogen and oxygen. The lines represent linear fits of the data.

S4 Gas-chromatograph calibration and baseline correction

The response of the gas chromatograph was calibrated by measuring the detector curve areas with different mixtures of calibration gases (5.0 purity) and a helium carrier gas stream. The

flow rate of helium was fixed at 30 mL/min, and the analyzed gas (O_2 and H_2) flow rates were varied to get concentrations between 1666 ppm and 11200 ppm. Once linearity in the GC response was confirmed in the range relevant to the experiments, further analysis was done on the basis of baseline measurements.

The baseline gas concentrations were recorded by generating gas in the cell described in SI Sec. S3 with Zirfon as the separator and two Pt coils as the electrode. These gas concentrations were taken to correspond to 100 % Faradaic efficiency. This is justified because of the absence of the buildup of a significant overpressure within the cell, and since the gases are generated far from the separator. Thus, the Faradaic efficiency of the PSi-ZGE was calculated as

$$\eta_F = \frac{a_{\text{GC}}}{a_{\text{baseline}}} * 100 \tag{2}$$

where a_{GC} and a_{baseline} are the areas under the detector curves for the experiment and the baseline measurement respectively. This method of measuring efficiencies was required because discrepancies were noticed in the oxygen concentration when absolute experiment detector curve areas were converted to concentrations. It was found that the up to 20 % of the O_2 was missing even in the absence of significant crossover, recombination or leaks. We ascribe this to the relatively high humidity of the gas streams from the electrolyzer.

S5 Cyclic voltammetry

Fig. SI 7 shows cyclic voltammograms of Pt-PSi-ZGE and Ni-PSi-ZGE.

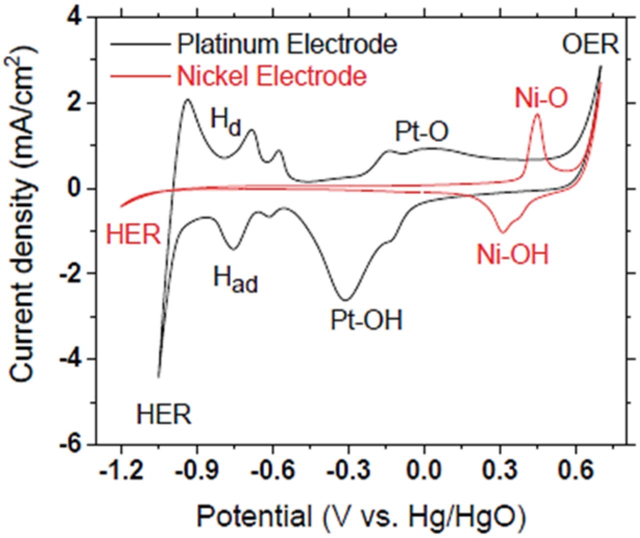

Figure SI 7: Cyclic voltammetry of platinum and nickel-coated Psi-ZGE.

S6 Bubble frequency analysis

Molar rate of hydrogen production

$$\dot{n}_{\mathrm{H}_2} = \frac{I}{zF} \tag{3}$$

Volumetric rate of hydrogen production

$$\dot{V}_{\mathrm{H}_2} = \frac{n_{\mathrm{H}_2} RT}{P} \tag{4}$$

Following the assumption that bubbles are spherical and have an average departure radius of $< r_d >$, we derive that the bubble departure frequency is,

$$f = \frac{\frac{RT}{P}\frac{I}{zF}}{\frac{4\pi}{3} < r_d^3 >} = \frac{3RT}{4\pi PzF}\frac{I}{< r_d >^3} \tag{5}$$

Therefore, for a given constant current, the frequency of bubble departure is $f \propto I \propto \frac{1}{<r_d>^3}$

S7 Flow cell design

Fig. SI 8 depicts the flow cell used to test the PSi-ZGEs. The flow cell is comprised of a pair of endplates machined out of polyoxymethylene (POM), two rubber gaskets, two stainless steel (SS316L) current collectors and a teflon spacer which holds the PSi-ZGE sample in place. The edges of the central cutout in the current collectors holds the PSi-ZGE sample in place and provides electrical conductivity. Flow channels are machined into the gaskets and the current collectors. Standard flat-bottom fluidic connectors were used to fasten tubing onto the end plates.

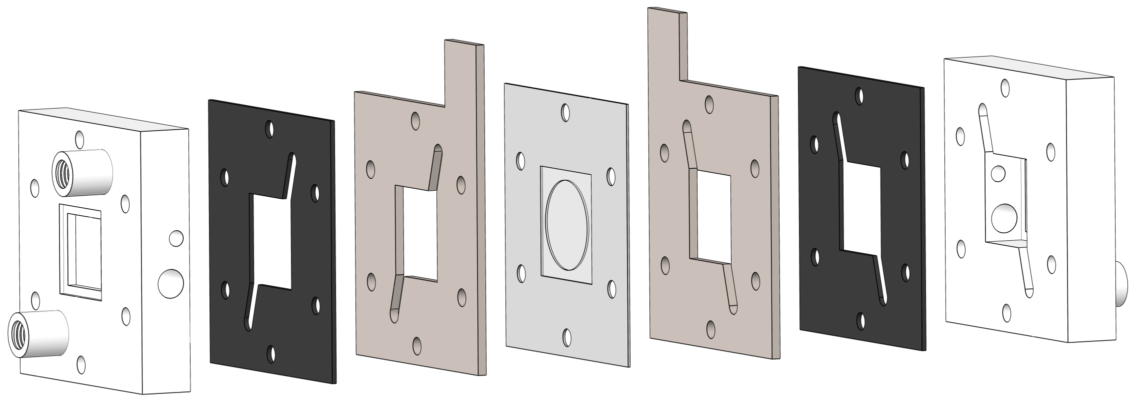

Figure SI 8: A schematic representation of the experimental set-up used to measure the performance of PSi-ZGEs with forced electrolyte convection

S8 SEM image after 24 h electrolysis

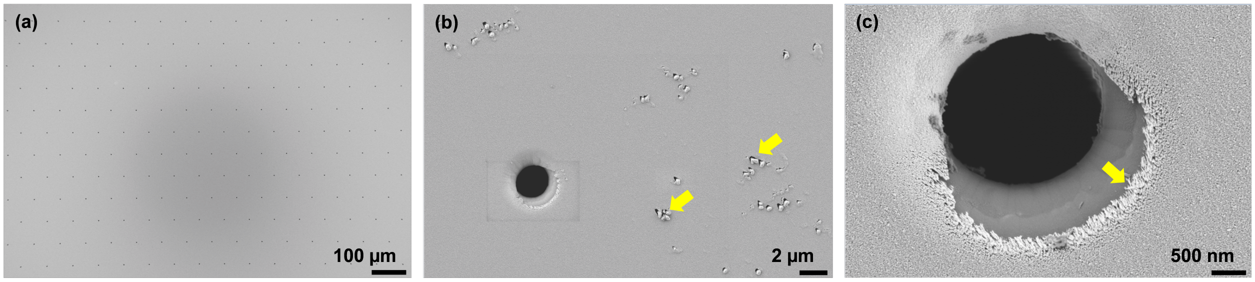

Figure SI 9: SEM images of a platinum-coated PSi-ZGE after 24 h of electrolysis at 100 mA/cm^2 at three different magnifications. Yellow arrows in (b) and (c) highlight areas where the roughness of the Pt film has increased.

The increase in the roughness of the platinum layer can be from physical degradation due to bubble nucleation and departure which can be mitigated by improving bubble removal pathways with optimized gas transport layers and electrolyte flow fields.

It is known from previous reports that the Pt surface undergoes a reconstruction under alkaline OER conditions[1] and it's likely to dissolve, albeit at slow rates[2]. This electrochemical degradation can be mitigated through the choice of more stable electrocatalysts. Since the electrocatalysts in the manuscript are deposited by chemical vapour deposition and employ the use of an adhesion layer, the thickness and material of the adhesion layer (Tantalum in this study) and the thickness of the electrocatalyst layer (200 nm Pt or Ni in this study) can be further optimized.

S9 Electrolyzer performance at 100 mL/min electrolyte flow rate

Porosity (%)	Current (mA)	Average potential (V)
7.7	50	1.73
7.7	100	1.89
7.7	250	2.26
7.7	500	2.67
7.7	800	3.22
1.69	50	1.82
1.69	100	1.99
1.69	250	2.48
1.69	500	3.38
1.69	800	4.45
0.6	50	2.09
0.6	100	2.29
0.6	250	2.80
0.6	500	3.51
0.6	800	4.43
0.15	50	2.01
0.15	100	2.37
0.15	250	3.47
0.15	500	> 5
0.15	800	> 5

Table SI 2: The average cell potential is tabulated for different applied currents and PSi-ZGE porosities at an electrolyte flow rate of 100 mL/min.

References

[1] M. Favaro, C. Valero-Vidal, J. Eichhorn, F. M. Toma, P. N. Ross, J. Yano, Z. Liu and E. J. Crumlin, *Journal of Materials Chemistry A*, 2017, **5**, 11634–11643.

[2] V. S. Bagotzky, E. I. Khrushcheva, M. R. Tarasevich and N. A. Shumilova, *Journal of Power Sources*, 1982, **8**, 301–309.
